# Supplementary material for: Loss of the Intellectual Disability and Autism Gene Cc2d1a and Its Homolog Cc2d1b Differentially Affect Spatial Memory, Anxiety, and Hyperactivity
Source: Front Genet. 2018 Mar 2;9:65. doi: 10.3389/fgene.2018.00065 (PMC5840150; doi:10.3389/fgene.2018.00065)
Supplement: Supplementary file 1 [file Image_1.PDF]

Supplementary Material

Loss of the intellectual disability and autism gene *Cc2d1a* and its homolog *Cc2d1b* differentially affect spatial memory, anxiety, and hyperactivity

Marta Zamarbide[1], Adam W. Oaks[1], Heather L. Pond[1], Julia S. Adelman[1], M. Chiara Manzini[1,2]

*** Correspondence:** Corresponding Author: M.C. Manzini: cmanzini@gwu.edu

1 Supplementary Figures and Tables

1.1 Supplementary Tables

Supplementary Table 1. Analysis of basic motor and sensory function in 1a-HET and 1b-HET mice

Genotype	Gender	Weight (g)	Righting reflex (s)	Wire hang (s)	Stride and gait	Tail pinch[a]	Visual reflex[b]
WT	M	28.39 ± 0.94	<1 s	56.5 ± 2.35	Normal	6/9	9/9
1b-HET	M	28.27 ± 0.77	<1 s	58.4 ± 1.10	Normal	8/9	9/9
1a-HET	M	27.97 ± 0.93	<1 s	55.5 ± 3.31	Normal	7/8	8/8
WT	F	21.06 ± 0.53	<1 s	58.7 ± 1.09	Normal	7/10	10/10
1b-HET	F	21.58 ± 0.50	<1 s	59.4 ± 0.38	Normal	8/10	10/10
1a-HET	F	20.40 ± 0.38	<1 s	59.36 ± 0.54	Normal	10/11	11/11

[a] Animals responding to tail pinch. [b] Animals responding to visual stimulus.

1.2 Supplementary Figures

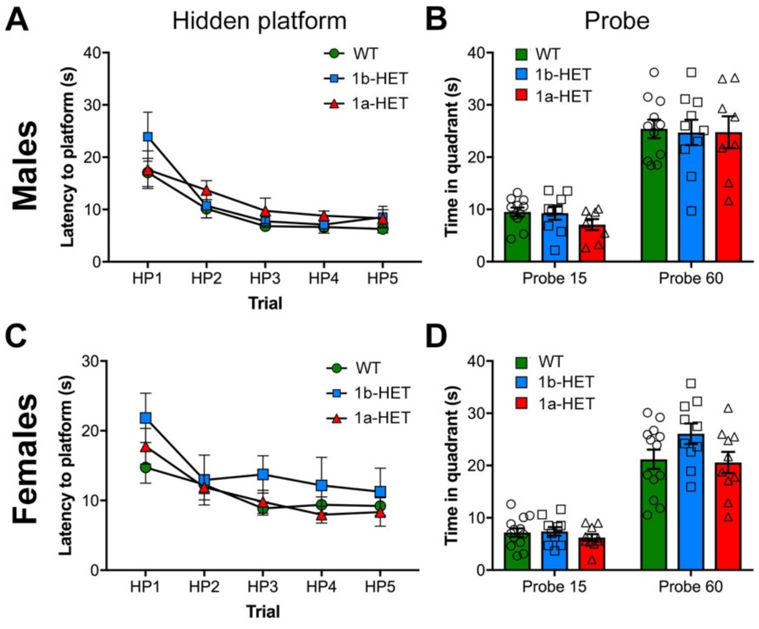

Supplementary Figure 1. *Cc2d1a* and *Cc2d1b* heterozygous mice do not show spatial memory deficits. Spatial learning and memory was studied by the Morris Water Maze test measured as latency to escape in three different stages, visible platform (VP; not shown), hidden platform (HP; **A** and **C**), or the reversal (RV; not shown) of the hidden platform location. During the probe trial the amount of time spent swimming where the platform was previously located was measured during 60s (**B** and **D**). No significant differences were found between *Cc2d1a* heterozygous mice (1a-HET, n=8 males; n=10 females), *Cc2d1b* heterozygous mice (1b-HET, n=10 males; n=10 females) and wild-type littermates (WT, n=11 males; n=13 females). **A-B.** Hidden platform trial (**A.**) and probe test at 15 and 60s (**B.**) for male animals. **C-D.** Hidden platform trial (**C.**) and probe test at 15 and 60s (**D.**) for female animals. Results expressed as mean ± SEM.

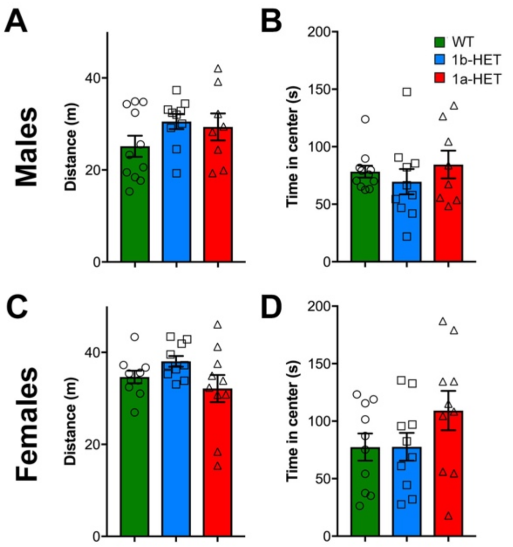

Supplementary Figure 2. *Cc2d1a* and *Cc2d1b* heterozygous mice do not show anxiety or hyper-activity. Exploratory and general locomotor activity in a novel environment was assessed in the open field test. **A-D.** No significant differences were found between *Cc2d1a* heterozygous mice (1a-HET, n=8 males; n=10 females), *Cc2d1b* heterozygous mice (1b-HET, n=10 males; n=10 females) and wild-type littermates (WT, n=11 males; n=13 females) in total path length (**A.** for males and **C.** for females) or time spent in the center of the apparatus (**B.** for males and **D.** for females). Results expressed as mean ± SEM.

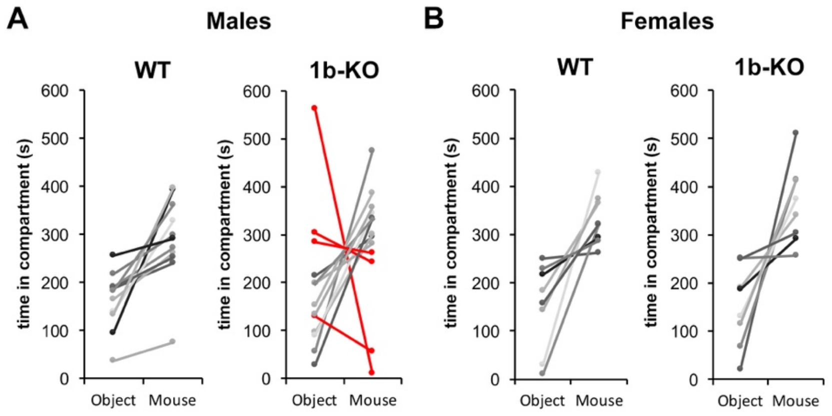

Supplementary Figure 3. Individual animal behavior for wild-type and *Cc2d1b* knock-out animals in three-chambered apparatus. A-B. Individual time spent with empty cup and stranger mouse for each test animals in wild-type (WT) and *Cc2d1b* knock-out (1b-KO) males (**A.**) and females (**B.**). 1b-KO males spending longer time with object vs. animal are shown in red.
